# 一个新的F10基因缺失突变所致遗传性凝血因子Ⅹ缺陷症家系及其致病机制研究

**DOI:** 10.3760/cma.j.cn121090-20240506-00169

**Published:** 2024-10

**Authors:** 栋彦 付, 晓梅 卢, 雅琳 于, 丽东 赵, 蕾 王, 嘉 杨, 嘉伟 郑, 端阳 王, 林花 杨, 刚 王

**Affiliations:** 山西医科大学第二临床医学院（第二医院）血液科，山西医科大学出凝血疾病及恶性血液病研究中心，血液病分子诊疗山西省重点实验室，太原 030001 Department of Hematology, The Sencond Hospital of Shanxi Medical University, Center for Shanxi Medical University and Tumor of the Hematopoietic and Lymphoid Tissues Diseases, Shanxi Provincial Key Laboratory for Molecular Diagnosis and Treatment of Hematological Diseases, Taiyuan 030001, China

**Keywords:** 凝血因子Ⅹ缺陷症, 基因突变, 凝血因子Ⅹ, 蛋白模型, Coagulation factor X deficiency, Gene mutation, Factor Ⅹ, Protein model

## Abstract

**目的:**

对一个新的F10基因缺失突变导致遗传性凝血因子Ⅹ（FⅩ）缺陷症家系进行基因突变分析，并研究其分子致病机制。

**方法:**

通过全外显子二代测序（NGS）筛选出先证者的F10基因突变位点，采用Sanger测序法进行验证。血液凝固法检测先证者及其家系成员的FⅩ活性，Sanger测序法分析其家系成员F10基因突变位点。采用Mutation Taster在线生物信息学软件预测突变的致病性；采用SWISS-MODEL软件模拟野生型和突变型三维蛋白模型分析突变对蛋白结构和功能的影响并对野生型和突变型FⅩ蛋白催化残基进行差异分析。通过构建质粒，转染人胚肾293T细胞（HEK 293T），RT-PCR方法分析突变位点的剪接情况，采用qRT-PCR的方法对F10基因mRNA水平进行定量分析，酶联免疫吸附试验（ELISA）方法分别检测细胞裂解液和细胞培养基（细胞内外）的FⅩ抗原水平。

**结果:**

血液凝固法检测先证者FⅩ活性为36.43%。NGS分析发现先证者F10基因第8号外显子携带一个缺失的杂合突变c.902_919del（p.Ala301_Glu306del），Sanger测序分析显示其家系部分成员（母亲与外祖父）也检测到相应的缺失突变。在线生物信息学软件预测显示c.902_919del突变为致病突变，致病分数为0.999；三维蛋白结构模型分析显示c.902_919del突变导致蛋白结构一段β折叠消失使得前一段β折叠长度减短，并且造成与其相邻氨基酸连接的氢键丢失，关键催化残基的侧链构象与野生型相比无明显差异。mRNA剪接分析显示该突变未发生选择性剪接改变，qRT-PCR结果显示表达c.902_919del突变型和野生型细胞中F10基因mRNA水平差异无统计学意义，ELISA结果显示突变型细胞培养基和裂解液的FⅩ∶Ag水平差异无统计学意义。

**结论:**

该遗传性FⅩ缺陷症家系由F10基因第8号外显子的c.902_919del（p.Ala301_Glu306del）杂合缺失突变导致。

## Abstract (en)

**Objective:**

To analyze the F10 gene mutations in a Chinese pedigree affected with the deficiency of the hereditary coagulation factor X（FX）, resulting from a new deletion mutation, and to study the associated molecular pathogenesis.

**Methods:**

Next generation sequencing（NGS）was performed to screen the genetic mutations in the proband which were then verified by Sanger sequencing. The FX activity（FX∶C）of probands and their family members was detected using the blood clotting method, and the mutation sites of the family members were analyzed using Sanger sequencing. The pathogenicity of the mutation site was predicted by using the online bioinformatics software, Mutation Taster. The SWISS-MODEL software was used for stimulating the three-dimensional models of the wild-type and mutant proteins for analyzing the influence of the mutation site on the structure and function of the proteins, and for analyzing the difference between the catalytic residues of the wild-type and the mutant proteins. The level of the F10 gene mRNA was quantitatively analyzed by qRT-PCR（quantitative reverse transcription polymerase chain reaction）method by constructing plasmids, transfecting human embryonic kidney 293T cells（HEK 293T）, and analyzing the splicing of the mutated site by RT-PCR method. The levels of FⅩ∶Ag in cell lysates and cell culture media（both inside and outside the cells）were detected by the ELISA（enzyme linked immunosorbent assay）method.

**Results:**

A medium-grade factor X deficiency with a 36.42% FⅩ∶C ratio was detected in the proband by the coagulation method. NGS analysis demonstrated a heterozygous deletion mutation in exon 8:c.902_919del（p.Ala301_Glu306del）in the proband. Sanger sequencing analysis indicated that some members of the family（mother and grandfather）were also carriers of the corresponding deletion mutation. Online bioinformatics software predicted the pathogenic nature of the c.902_919del mutation, with a pathogenic score of 0.999. The 3D protein structure model analysis indicated that the c.902_919del mutation resulted in the disappearance of a segment of β-fold in the protein structure, thereby shortening the preceding segment of the β-fold and a subsequent loss of hydrogen bonds between adjacent amino acids with no significant difference in the side chain conformation of the key catalytic residues compared to the wild-type. mRNA splicing analysis indicated the absence of alternative splicing changes in the mutation, and qRT-PCR results indicated the absence of a statistically significant difference between the mRNA levels of F10 gene and wild-type mRNA in cells expressing c.902_919del mutant. The ELISA results indicated that there was no statistically significant difference in the FX∶Ag levels of the mutant cell culture medium and the lysate.

**Conclusion:**

In this pedigree, the heterozygous mutation in exon 8 of F10 gene（c.902_919del, p.Ala301_Glu306del）caused the hereditary factor Ⅹ deficiency.

遗传性凝血因子Ⅹ（FⅩ）缺陷症是一种由于F10基因突变而导致FⅩ抗原（FⅩ∶Ag）含量减少或活性（FⅩ∶C）降低的常染色体隐性遗传性出血性疾病。根据流行病学推算，FⅩ缺陷症约占罕见出血疾病的5.9％，患病率为1/100万[1]。FⅩ是一种维生素K依赖的血浆丝氨酸蛋白酶，是凝血酶生成共同途径中的一种酶，在凝血共同途径中起重要作用[2]。FⅩ既可以被FⅨa/FⅧa内源性凝血途径激活，又可以被FⅦa/TF外源性凝血途径激活，最终转变为激活形式的FⅩ（FⅩa）[3]。FⅩa可与激活的FⅤa、磷脂酰丝氨酸以及Ca^2+^形成凝血酶原复合物，促进凝血酶原转化为凝血酶[4]，也是凝血途径的关键。F10基因位于染色体13q34，包含8个外显子和7个内含子，编码488个氨基酸。FⅩa在肝脏中合成并分泌到血浆中（血浆浓度为8～10 µg/ml）。血浆FⅩ是由通过二硫键连接的17 kDa轻链和45 kDa重链组成的双链蛋白[5]。作为丝氨酸蛋白酶，FⅩ轻链中含有Gla结构域和两个表皮生长因子（EGF）结构域，重链中含有催化结构域，FⅩ的活性催化三联体由位于重链的His276、Asp322和Ser419组成[6]。FⅩ与其他维生素K依赖性蛋白质（FⅦ、FⅨ、凝血酶原、蛋白S和蛋白C）同源[7]。FⅩ缺陷症的主要确诊依据是临床表现及实验室检查凝血酶原时间（PT）、活化部分凝血活酶时间（APTT）延长以及FⅩ∶C明显降低，FⅩ∶C降低与出血的严重程度有关[8]。FⅩ∶C＜10％为重型，一般会发生严重的自发性出血；FⅩ∶C 10％～40％为中型，会出现轻微的自发性出血；FⅩ∶C＞40％为轻型，自发性出血风险较低[9]。有报道显示，即使杂合突变患者也可能发生FⅩ∶C 40％～60％伴轻度出血[10]。FⅩ缺陷症的临床表现极具特异性，鼻出血是FⅩ缺陷症最常见的症状[11]，也可发生关节/软组织出血、月经量多及胃肠道出血，重型患者可能合并中枢神经系统出血和手术后出血[12]。F10基因突变数据库已登记的遗传性FⅩ缺陷症相关F10基因突变有180多种，突变类型包括无义突变、错义突变、缺失突变和插入突变等。

本研究中，我们报告一个遗传性FⅩ缺陷症家系并研究其分子发病机制。

## 对象与方法

1. 家系资料：先证者为女性，12岁，2022年7月因腿部不明原因出现瘀点、瘀斑就诊于山西医科大学第二医院血液内科门诊。实验室检查：APTT、PT延长（APTT 42.40 s、PT 18.20 s），FⅩ∶C为36.43％，其余凝血因子活性均正常，血管性血友病因子（VWF）抗原正常。先证者的父母为非近亲婚配，无自发性出血倾向。本研究经山西医科大学第二医院伦理委员会批准（2023YX第159号），受试者均签署知情同意书。

2. 样本收集与处理：采集先证者及其家属枸橼酸钠抗凝外周血2管，每管约2.5 ml。在室温下1 500×*g*离心15 min，分离血浆，使用STA compact max（法国STAGO公司）检测FⅩ∶C，使用ELISA法检测先证者及其家系成员的FⅩ∶Ag水平。另外一管−80 °C冻存，用于提取基因组DNA。

3. 二代测序和基因组DNA提取：先证者血样由上海派森诺生物科技有限公司进行高通量全外显子NGS测序，获得可疑的变异位点。使用D3396 Blood DNA Mini Kit试剂盒（美国Omega TMM公司）提取先证者及其家属全血基因组DNA。使用CYT3MF多功能酶标仪（美国BioTek公司）检测DNA浓度和纯度，用于下一步PCR扩增。

4. PCR扩增和Sanger测序：根据NCBI数据库（https://www.ncbi.nlm.nih.gov/）提供的F10基因序列（GeneID:2159；NC_000013.11）和NGS测序获得的可疑变异位点，采用SnapGene v6.0.2软件根据突变位点设计引物（表1）。引物由北京六合华大基因科技有限公司合成。PCR体系为50 µl。包含Premix TaqTM（TaKaRa TaqTM Version 2.0 plus dye，货号RR901A，日本TaKaRa公司）25 µl，基因组DNA 6 µl，ddH_2_O 15 µl，正向引物（F10-01F）、反向引物（F10-01R）各2 µl。PCR反应条件：94 °C预变性5 min；94 °C变性30 s，57 °C退火30 s，72 °C延伸30 s，共设置35个循环；72 °C延伸10 min；4 °C保存。PCR结束后将5 µl的PCR扩增产物通过2％的琼脂糖凝胶电泳进行验证。将PCR产物送至北京六合华大基因科技有限公司进行Sanger测序。测序结果与NCBI数据库所提供的F10基因序列在SnapGene软件上进一步分析，以确定开始发生缺失的变异位点和缺失的范围。通过检索HGMD人类基因突变数据库（http://www.hgmd.cf.ac.uk/ac/index.php）、CliVar临床遗传变异数据库（https://www.ncbi.nlm.nih.gov/clinvar/）、单核苷酸多态性SNP数据库（https://www.ncbi.nlm.nih.gov/snp/）和EAHAD F10数据库（https://f10-db.eahad.org/）对该缺失的突变位点信息进行检索。

**表1:** F10基因PCR扩增引物名称及序列

引物名称	引物序列（5′→3′）	片段大小（bp）
F10-01F	AACTATTTTACACTCTCAGCCAGCG	474
F10-01R	GGCGATGTCGAAGTCATAGGTCTC	
F10-PD-F	GATTCAAGGTGAGGGTAGGTAAGTGAC	1 546
F10-PD-R	ACGGCGATGTCGAAGTCATAGGTC	
F10-ZT-F	GGAGACCTATGACTTCGACATCGC	6 834
F10-ZT-R	ACCTACCCTCACCTTGAATCTCTTG	
F10-ZL-F	GTTTCTGTGGTGGAACCATTCTGAG	589
F10-ZL-R	ACCTTGGTGTAGATCCCGTACTTC	571

5. 生物信息学分析：应用MutationTaster（http://www.mutationtaster.org/）在线软件评估缺失突变位点的致病性。

6. 质粒表达载体的构建：采用pCMV-F10（human）-3×FLAG-Neo作为载体模板（购买于武汉淼灵生物技术有限公司），设计与相邻载体片段末端具有重叠序列的扩增引物，使用引物（F10-PD-F、F10-PD-R）通过PCR扩增获得目的片段，使用引物（F10-ZT-F、F10-ZT-R）通过反向PCR扩增将载体切开，采用无缝克隆的技术构建质粒（图1），通过转化感受态细胞，获得野生型质粒（F10-WT）和突变型质粒（F10-MT）。

**图1:**
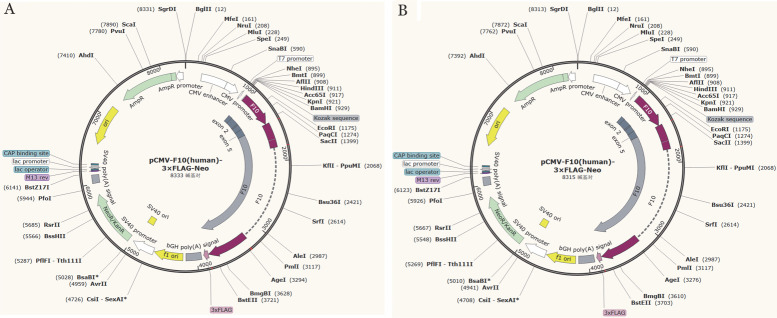
F10基因质粒构建图 **A** 野生型；**B** 突变型

7. F10 mRNA体外剪接及表达测定：由于FⅩ在肝脏中特异性表达，因此无法从外周血提取RNA来分析该突变的剪接情况[13]，所以本研究通过构建质粒（图1），将F10-WT和F10-MT和表达GFP质粒分别转染HEK293T细胞。转染后培养48 h，收集细胞提取RNA，通过逆转录合成cDNA，设计引物F10-ZL-F、F10-ZL-R（表1），采用RT-PCR的方法分析野生型及突变型细胞F10 mRNA的剪接情况，采用qRT-PCR的方法对F10 mRNA的表达水平进行定量分析，采用ELISA方法分别检测细胞裂解液和细胞培养基（细胞内外）的FⅩ：Ag水平。

8. FⅩ蛋白三维结构及其空间模拟构型分析：采用SWISS-MODEL软件同源建模FⅩ野生型和FⅩ突变型蛋白的三维结构，使用PyMol软件对蛋白三维结构图进行可视化编辑分析并对野生型蛋白和突变型蛋白催化残基进行差异分析。

## 结果

1. FⅩ∶C和FⅩ∶Ag检测结果：先证者、母亲和外祖父FⅩ∶C均偏低，其余家系成员FⅩ∶C在正常参考范围。ELISA法检测到先证者FⅩ∶Ag水平接近正常，其余家系成员FⅩ∶Ag水平正常。详见表2。

**表2:** 家系成员凝血因子Ⅹ活性（FⅩ∶C）和抗原（FⅩ∶Ag）检测结果（％）

家系成员	PT法FⅩ∶C	APTT法FⅩ∶C	FⅩ∶Ag
先证者	36.43	40.87	67.4
父亲	96.60	85.49	125.1
母亲	71.27	62.25	85.3
外祖父	48.66	35.65	77.9
外祖母	72.17	73.27	93.9

参考值范围	70.00~120.00	70.00~120.00	70.0~120.0

**注** PT：凝血酶原时间；APTT：活化部分凝血活酶时间

2. F10基因测序分析：NGS测序显示，先证者F10基因第8号外显子上存在杂合缺失突变c.902_919del（p.Ala301_Glu306del），并经Sanger测序验证（图2）。通过家系基因分析，先证者的母亲和外祖父均存在该缺失突变，其父亲、祖母与外祖母为野生型（图3）。查询国内外数据库，未见c.902_919del（p.Ala301_Glu306del）突变的相关报道。

**图2:**
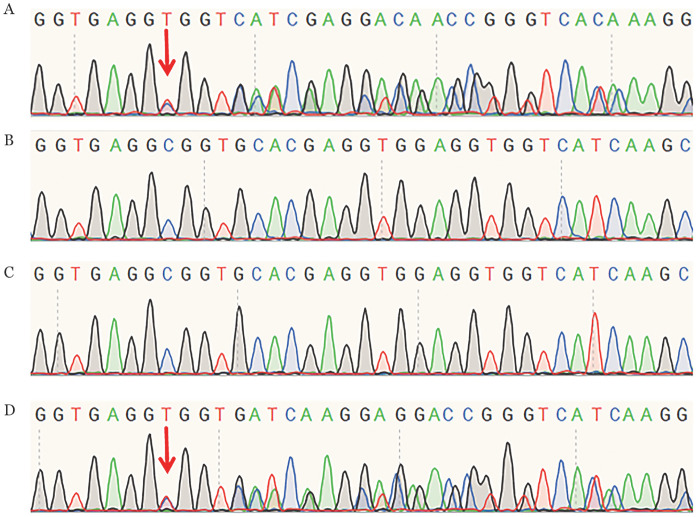
凝血因子Ⅹ缺陷家系成员Sanger测序结果 **注** **A** 先证者；**B** 野生型；**C** 先证者父亲；**D** 先证者母亲。箭头所示为开始发生突变的位点

**图3:**
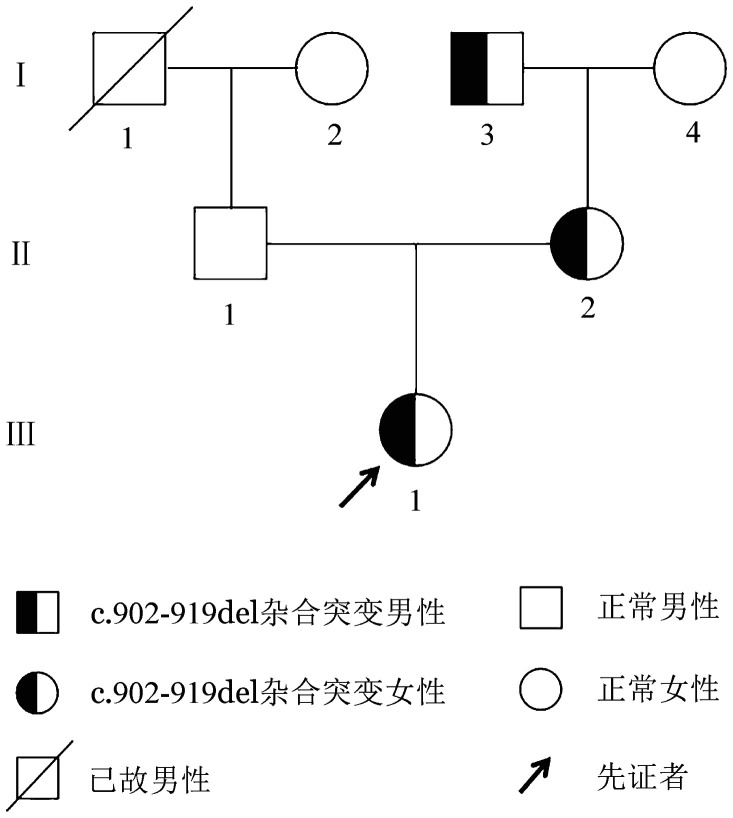
遗传性凝血因子Ⅹ缺陷症家系图

3. 生物信息学分析结果：应用MutationTaster对该突变进行致病性评估，结果显示“disease causing”，致病评分为0.999，并提示可能存在氨基酸结构改变，蛋白功能受影响以及该突变可能导致缺失突变位点后第5个碱基处发生选择性剪接。

4. F10基因mRNA体外剪接及表达测定结果：通过构建质粒（图1）分别转染HEK 293T细胞，采用RT-PCR方法分析其剪接结果显示野生型和突变型条带均为单一条带，并未出现预测的选择性剪接位置的条带（图4A），Sanger测序结果显示该突变并未造成选择性剪接（图4B）。通过三个独立测试的qRT-PCR结果显示，野生型和突变型mRNA水平分别为（1.06±0.56）、（1.12±0.42）（*t*＝0.16，*P*＝0.88），表明c.902_919del突变不影响细胞内mRNA的转录。ELISA结果显示突变型细胞培养基和裂解液的FⅩ∶Ag分别为（110±34）％、（106±42）％（*t*＝0.35，*P*＝0.74），突变型细胞培养基和裂解液的FⅩ∶Ag水平差异无统计学意义。

**图4:**
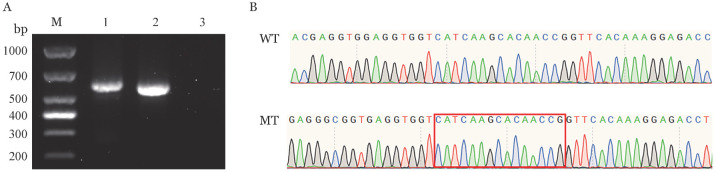
野生型（WT）及突变型（MT）F10 mRNA体外剪接及表达测定结果 **注** **A** RT-PCR扩增电泳结果图，M：DNA Marker；1：WT；2：WT；3：GFP阴性对照；WT条带扩增片段为589 bp，MT条带扩增片段为571 bp。**B** Sanger测序结果，方框内为预测发生选择性剪接的位置

5. FⅩ蛋白三维结构及其空间模拟构型分析结果：c.902_919del突变相对应的导致301-306位点的氨基酸缺失。应用PyMol软件分析表明，该缺失突变改变了蛋白的结构构象，导致一段β折叠消失使得前一段β折叠长度减短，导致本来与其附近氨基酸所相连的氢键丢失，并缺失了氨基酸之间的相互作用力（图5）。对关键催化残基His276、Asp322和Ser419（图6）进行分析发现，当突变型蛋白与底物结合后，关键催化残基的侧链构象与野生型相比无明显差异。

**图5:**
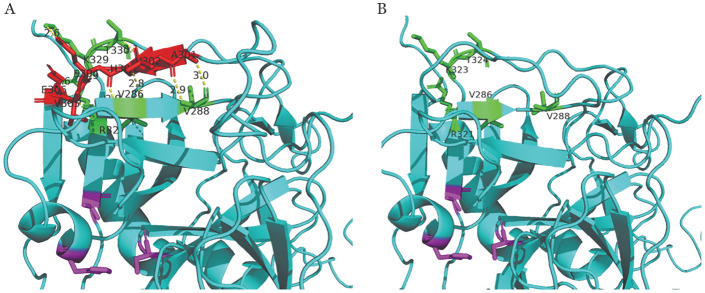
野生型（A）和突变型（B）凝血因子Ⅹ（FⅩ）蛋白三维结构图 **注** 蓝色背景表示FⅩ蛋白的结构，红色部分为301-306氨基酸位点A301、V302、H303、E304、V305、E306，绿色部分为与其存在氢键作用力相连的5个氨基酸位点V286、V288、R327、K329、T330，黄色虚线为氢键作用力，紫色部分为FⅩ蛋白催化三联体的关键氨基酸His276、Asp322和Ser419

**图6:**
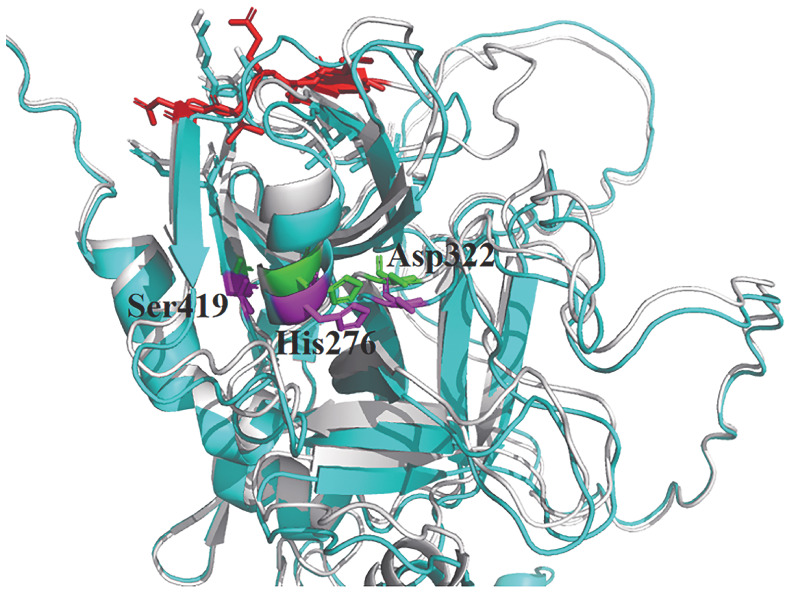
野生型与突变型凝血因子Ⅹ蛋白催化残基的差异分析 **注** 灰色背景表示野生型蛋白；蓝色背景表示突变型蛋白；绿色棒状标识是野生型蛋白与底物结合后的催化残基构象；紫色棒状标识是与突变型蛋白与底物结合后的催化残基构象

## 讨论

FⅩ缺陷症属于一种罕见的常染色体隐性遗传性出血疾病，是严重的凝血因子缺陷症之一，仅次于血友病A和血友病B[14]。大多数常染色体基因是双等位基因，然而在哺乳动物中也有一些正常的单等位基因表达，其中包括仅遗传自母本或父本的非随机表达的印迹基因，以及在常染色体基因中的各种随机单等位基因表达[15],[16]。女性血友病是X染色体隐性遗传，传统上认为单杂合突变的血友病只有男性为患者，女性为携带者。近几年国际血栓与止血学会（ISTH）对于女性血友病进行了新的命名：FⅩ∶C<40％为女性血友病患者，>40％为女性血友病携带者，根据携带者是否存在出血症状又分为有症状的携带者和无症状的携带者[17]。FⅩ缺陷症在临床上可分为Ⅰ型（交叉反应物质阴性，FⅩ∶C和FⅩ∶Ag水平均较低）和Ⅱ型（交叉反应物质阳性，FⅩ∶C水平异常，FⅩ∶Ag水平正常或接近正常）[18]。与其他维生素K依赖性凝血因子缺乏症不同，FⅩ缺乏症的症状主要是出血，如易擦伤、血肿、鼻出血、关节积血、颅内出血、胃肠出血等[19]。由于FⅩ在凝血级联反应中起着关键作用，因此FⅩ缺陷出血风险高于其他凝血因子缺陷[20]。F10基因的外显子1编码信号肽区域，外显子2编码前肽及甘氨酸区，外显子3编码芳香族氨基酸区，外显子4编码第1个EGF区，外显子5编码第2个EGF区，外显子6编码活化肽，外显子7和8编码催化区[21]。FⅩ缺陷最常见的突变位点位于外显子7和8的FⅩ催化位点以及位于外显子2的谷氨酸结构域[22]。并且大多数发生在第8号外显子，这可能与它是F10基因中最大的外显子有关[23]，也可能与第8号外显子的催化结构域相关，包含催化位点His236、Asp282和Ser379，这些位点对FⅩ的催化能力至关重要。催化结构域的突变往往对蛋白质的结构和功能有重要影响。Nagaya等[24]研究发现p.Gly263Val和p.Val424Phe影响突变蛋白折叠；p.Arg387Cys降低FⅤa结合亲和力，p.Gly406Ser抑制FⅩa与凝血酶原的相互作用。Tartary等[25]研究证明，血友病B中高度保守的氨基酸取代（Ser308Asn）可能是丝氨酸蛋白酶的重要组成部分，从而导致轻度表型。因此，在空间上氨基酸接近301-306位点的其他突变可能有助于我们理解发生的机制[26]。

在本研究中，我们分析了一个FⅩ缺陷症先证者，其近期发现皮肤有异常出血，APTT和PT均延长，FⅩ∶C较低，FⅩ∶Ag水平接近正常，属于Ⅱ型FⅩ缺陷症。我们收集患者外周血，并提取基因组DNA，应用NGS技术对该患者进行基因诊断。遗传分析显示患者存在小片段的杂合缺失突变c.902_919del（p.Ala301_Glu306del），后续通过对患者家属的血液样本进行研究，发现其母亲和外祖父具有相同的突变情况，母亲APTT法FⅩ∶C偏低，FⅩ∶Ag水平正常，外祖父FⅩ∶C较低，FⅩ∶Ag水平正常，他们的表型也符合Ⅱ型FⅩ缺陷症。后期通过回访了解到先证者平日有频繁性鼻出血的表现，而母亲并无任何出血症状，外祖父无自发性出血，但止血时间会相对正常人延迟。尽管三人具有同样的突变，但由于人体之间存在很大的个体差异性，所以导致出血表现不同。纽约基因组中心将这种现象称为可变外显率，是指致病变异的严重程度在个体之间不同[27]。生物信息学软件预测，结果显示该缺失突变致病的分数极高，可能会造成氨基酸结构功能的改变以及会发生剪接位点的改变。为了研究该突变如何改变剪接位点情况，我们通过构建野生型和突变型质粒，并将质粒分别转染到HEK293T细胞中，从细胞中提取RNA，结果该突变并未造成剪接位置的改变，这可能与预测剪接位点改变的分数仅仅为0.39有关。通过对F10基因mRNA的表达水平进行定量发现突变型c.902_919del转录水平没有降低。ELISA结果显示突变型细胞培养基和裂解液的FⅩ∶Ag差异不显著。FⅩ蛋白晶体结构分析表明，活性催化三联体定位于His276、Asp322和Ser419残基，三维模拟结构图可明显看出该突变导致蛋白结构的改变，并导致与相邻氨基酸的相互作用力消失。虽然其与相邻的氨基酸在空间结构上都靠近底物结合位点，但该缺失并未影响活性位点的形状及其对FⅩ蛋白的作用。因此，我们认为该缺失突变不影响底物的结合，不会造成蛋白质功能障碍。

综上所述，本研究通过对一个遗传性FⅩ缺陷症家系进行表型及基因分析，发现F10基因第8号外显子新的杂合缺失突变c.902_919del（p.Ala301_Glu306del）是该家系的发病原因。
